# Biopriming of Durum Wheat Seeds with Diazotrophic Bacteria Improved Physiological and Biochemical Pathways Under Salt Stress

**DOI:** 10.3390/ijms27198889

**Published:** 2026-10-07

**Authors:** Adel Hadj Brahim, Zouhour Said, Eya Frikha, Mouna Jlidi, Manel Ben Ali, Mamdouh Ben Ali

**Affiliations:** 1 Laboratory of Microbial Biotechnology, Enzymatic, and Biomolecules (LMBEB), Centre of Biotechnology of Sfax (CBS), University of Sfax, P.O. Box 1177, Sfax 3018, Tunisia; 2 Astrum Biotech, Business Incubator, Center of Biotechnology of Sfax (CBS), University of Sfax, Road of Sidi Mansour km 6, P.O. Box 1177, Sfax 3018, Tunisia

**Keywords:** seed biopriming, PGPB, proline, CAT, APX, salt stress, *Triticum turgidum* L. var *durum*

## Abstract

A significant strategy for sustainable management, by decreasing the use of chemical fertilizers, can consist of technology based on suitable levels of plant growth promoting bacteria (PGPB) seed biopriming. The main goal of this work was to demonstrate the positive effect of PGPB as seed biopriming factor on the growth of durum wheat plantlets under stress. plantlets physiological and biochemical pathways modifications were assessed in controlled growth chamber under salt stress. Seeds bioprimed with bacterial strains, in particular, *Bacillus pumilus* (MA9), *Virgibacillus halodenitrificans* (MA14), *Bacillus subtilis* (MA17), and *Bacillus pumilus* (MA19), demonstrated the best development of durum wheat seedlings under salt stress. The total length and total dry weight were altogether higher in bioprimed than in unbioprimed plants. The PGPB-bioprimed plants were very sound and hydrated, and the unbioprimed plant leaves were parched within 120 mM NaCl. In contrast to explants without PGPB, all the biochemical variables, for example, auxin, proline, soluble sugars, amino acids, and protein, were essentially improved in bioprimed explants. Biopriming with MA17 fundamentally diminished the reactive oxygen species (ROS) rate, particularly without salt stress. In any case, this impact of the hindrance of “ROS” was more pronounced under stressful conditions. The explant antioxidant prevention agent pathways catalase (CAT) and ascorbate peroxidase (APX) and the chlorophyll content were improved after seed biopriming with PGPB when explants with unbioprimed seeds had low cell reinforcement pathways under the two treatments, particularly under pressure. The bioprimed plants additionally had higher K^+^/Na^+^ proportions and expanded intracellular K^+^ ion activity. Hence, it can be anticipated that seed biopriming with PGPB is a novel technology that can improve healthy plant development under saline conditions. This study shows that PGPB has a significant effect in initiating resistance under stressful conditions in plants and can be utilized to develop new bioinoculants to reduce the use of chemical fertilizers.

## 1. Introduction

Plants are exposed to various environmental stresses during growth and development under natural and agricultural conditions. How adverse environments affect plant growth is not only a fundamental scientific question but is also of vital importance for agriculture and food security. Several strategies to alleviate stress-induced adverse effects on plant growth have been described in many comprehensive reviews [1,2,3]. For example, high levels of ethylene that were produced under stressful environments can be reduced by the application of various ethylene inhibitors, such as amino ethoxy vinyl glycine (AVG), cobalt ions (Co^2+^), and silver ions (Ag^+^), leading to the enhancement of plant growth by alleviating the adverse effects of high levels of ethylene [4]. However, these agrochemicals are expensive and toxic to humans and soil health [5]. Unexpected environmental effects have resulted from the extensive use of chemical fertilizers to address nutrient deficiencies and maximize agricultural productivity [6]. Because of the severe issues with managing the water and nutrient supply at the moment, farmers use large amounts of fertilizers to maximize profits, even if this lowers the quality of the soil and groundwater [7]. Furthermore, despite increasing crop yields, the use of such chemicals results in a diminished net cash return for farmers. There are also environmental concerns about the persistence of these chemicals in the soil environment [8,9]. The other negative impacts of stress, such as specific ion toxicity caused by salinity or root desiccation under drought stress, may not be overcome by using these chemicals. Although plants employ some specific mechanisms to combat these stresses, beneficial microbial populations in the rhizosphere also play a significant role in reducing the intensity of stress [10]. Various scientific research works have focused on developing technological approaches for modifying saline soil by rehabilitation measures or adopting biotic approaches using salt-tolerant plant crops, which is not an easy and cost-effective approach for sustainable agriculture [11]. In this case, it is urgent to develop appropriate techniques for improved cereal production capacity in inefficiently cultivated or uncultivated areas, such as seed biopriming with diazotrophic PGPB [12]. The use of microbial vectors, which can reduce salt stress and encourage plant growth and control diseases, is a promising strategy for enhancing plant growth in salinated soils [12,13,14,15]. The application of plant-growth-associated bacteria that engage with plants to alleviate stress is a novel, advanced method for addressing salinity. Numerous studies have shown that beneficial bacteria can improve plant resilience to unfavorable environmental stressors, such as drought, salt, nutrient inadequacy, and heavy metal contamination. Under stressful circumstances, these inoculants aid in the establishment of sustainable agriculture [16,17,18]. Inoculation of seeds of various crop plants (such as tomato, pepper, canola, bean, and lettuce) with PGPB, can ameliorate the plant tolerance to high levels of salt and lead to enhancement of plant growth by increasing root and shoot growth, dry weight, fruit and seed yields [13,19]. The ability of seed biopriming to promote seed germination and offer early assistance for seedling growth under stressful circumstances has garnered a lot of interest. Utilizing a variety of microbes and microbe-mediated signaling molecules, seed biopriming strategies include seed coating, pelleting, hardening, and other biological seed germination improvement methods [20]. The seed can be penetrated by natural microbial communities that are connected to its exterior space. Imbibition time is shortened when hydration effectiveness is increased, which raises the germination rate [21]. It has previously been demonstrated that using microbial inoculants containing plant growth-promoting bacteria (PGPB) can boost crop yields by 12–20% and that biopriming seeds with PGPB and other beneficial bacteria improves plant efficacy and resilience in challenging environments. [22].

The general impacts of salinity stress on plant growth include hormonal and nutritional imbalance, physiological disorders such as epinasty, abscission, and senescence, and susceptibility to diseases [23,24,25]. Moreover, under salinity stress, ion toxicity occurs, particularly due to excessive amounts of Na^+^ and Cl^−^, which cause harmful effects on plant growth and development [26,27]. Certain abnormalities may occur in plants under stress, and their intensity increases under such conditions. Plants adopt specific strategies to overcome the negative impact of stress. In most stressful environments, reactive oxygen species (ROS), such as superoxide, hydrogen peroxide, and hydroxyl radicals, which are detrimental to normal plant growth and development, are produced [28,29]. The presence of ROS can cause cellular damage through the oxidation of lipids and proteins, chlorophyll bleaching, and damage to nucleic acids, ultimately leading to cell death [30,31]. Plants develop self-defense mechanisms by producing antioxidant enzymes, such as superoxide dismutase, ascorbate peroxidase, glutathione reductase, and catalase [32,33]. In a saline environment, in addition to nutritional and hormonal imbalances, plant water uptake decreases due to changes in soil water potential [34]. Under such conditions, the accumulation of compatible solutes, namely, proline, glycine betaine, trehalose, polyols, and many others, takes place in the plant body [35,36]. This phenomenon plays an important role in protecting the plant from stress-induced deleterious effects by osmotic adjustment, limiting water loss, and diluting the concentration of toxic ions [37]. The accumulation of compatible solutes enables plants to maintain their osmotic potential for enhanced uptake of water.

In the present study, four *Bacillus* strains were isolated from the roots of the Halophyte *Salicornia brachiata* growing in coastal saline soil in Tunisia (34°46′16″ N 10°48′24″ E). The isolates were checked for improvement of salt stress tolerance of durum wheat under natural and saline conditions. After one month of hydroponic culture, the plants were also tested for physiological and biochemical aspects such as auxin content and lipid peroxidation.

## 2. Results

### 2.1. Biochemical Characterization of PGPB Strains

**Figure 1:**
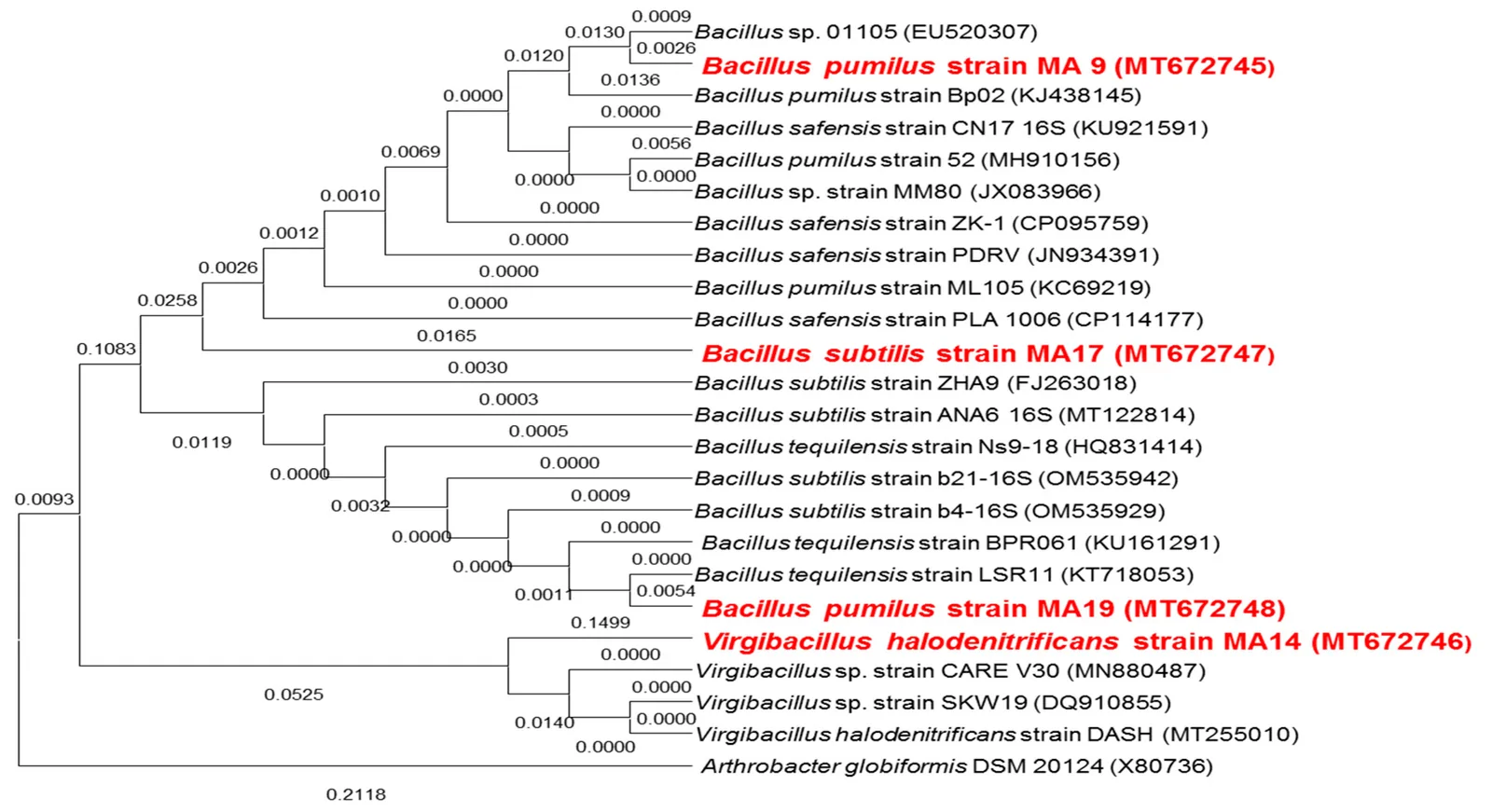
Distance matrix tree based on 16S rDNA gene sequence alignment of selected PGPB bacteria marked in red: *Bacillus pumilus* strain MA9; *Virgibacillus halodenitrificans* strain MA14; *Bacillus subtilus* strain MA17, and *Bacillus pumilus* strain MA19, and other closely related bacteria.

All PGPB strains used for seed biopriming of durum wheat were identified based on the 16S RNA gene [12] and the Tree of the distance matrix and Distances were calculated using Pairwise Sequence Alignment using Blast, and trees were constructed using the Neighbor Joining method (Figure 1). The screening results for PGP traits are duplicated in Table S1. The growth capacity under different salt concentrations was studied after 48 h of incubation at 30 °C (Figure S1). All selected strains were able to grow under 125 mM NaCl, beyond which *Bacillus pumilus* strain MA9 and *Virgibacillus halodenitrificans* strain MA14 underwent a significant decrease, while the two strains, *Bacillus subtilis* strain MA17 and *Bacillus pumilus* strain MA19 persisted with significant growth. IAA production (Figure S2), phosphate solubilization (Figure S3), siderophore production and antifungal activity were observed in all isolates of PGPB. All isolates were negative for HCN (Table S1) but positive for ammonia production, EPS secretion, and ACC deaminase production (Table S1). They were tested for the quantitative estimation of IAA in the presence and absence of tryptophan. The results showed that with the addition of tryptophan, the production of IAA was higher than with the addition of no tryptophan for all strains in LB medium (Figure S2). With the addition of tryptophan, the production of IAA increased, which, along with phosphate solubilization, was highest in *Virgibacillus halodenitrificans* strain MA14 and *Bacillus pumilus* strain MA9, whereas *Bacillus subtilis* strain MA17 and *Bacillus pumilus* strain MA19 had equal proportions of phosphate and IAA production (Figures S2 and S3).

**Figure 2:**
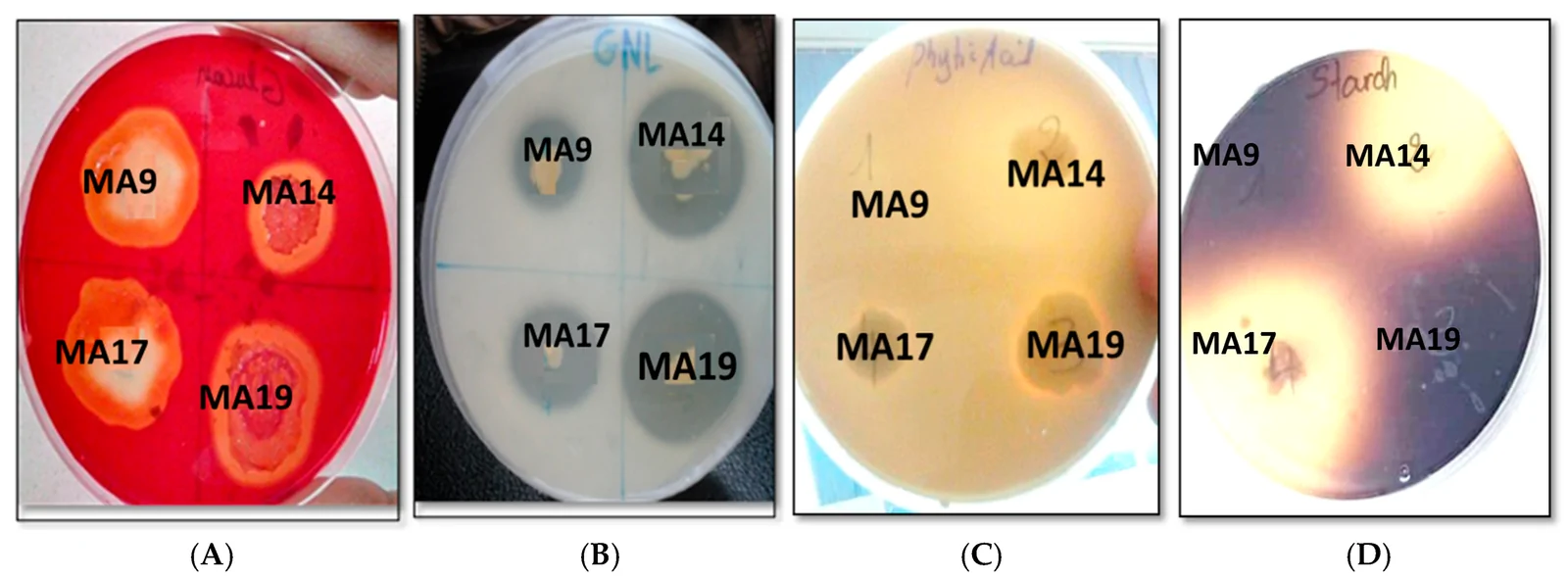
Production of β-glucanase (**A**), proteases (**B**), phytases (**C**) and amylases (**D**) in vitro by bacterial culture of: 1: *Bacillus pumilus* strain MA9, 2: *Virgibacillus halodenitrificans* strain MA14, 3: *Bacillus subtilis* strain MA17 and 4: *Bacillus pumilus* strain MA19 after 7 days at 28 ± 2 °C. The formation of a halo around the culture shows a positive activity. Scale bars 50 µm.

**Figure 3:**
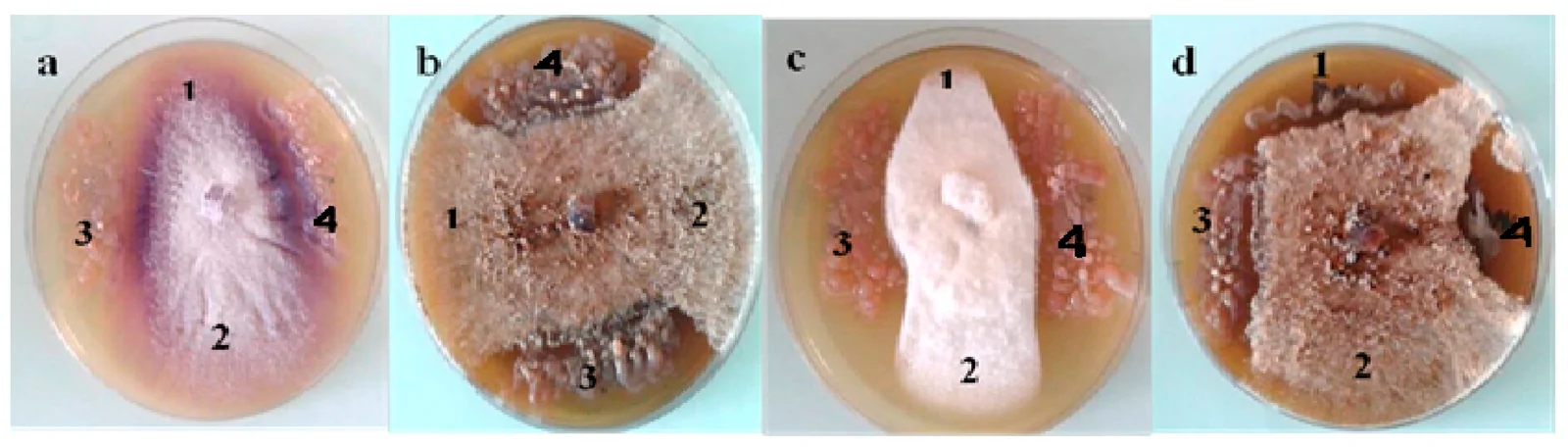
Biocontrol of selected strains 1: *Bacillus pumilus* strain MA9; 2: *Virgibacillus halodenitrificans* strain MA14; 3: *Bacillus subtilis* strain MA17; 4: *Bacillus pumilus* strain MA19, against (**a**) *Fusarium oxysporum*, (**b**) *Fusarium graminearum*, (**c**) *Fusarium solani*, and (**d**) *Rhizoctonia solani*. Scale bars 50 µm.

All the tested PGPB were found to produce phytase, β-glucanase, protease, and amylase activities (Figure 2). The antifungal activity of all strains was checked against *Fusarium solani*, *F. oxysporum*, *F. graminearum* and *Rhizoctonia solani* using PDA medium (Table S1). The antifungal activities of the tested strains varied according to PGPB and phytopathogenic fungal strains. In fact, *Bacillus subtilis* strain MA17 is the most effective against all fungal strains, and it is the only efficient strain against *Fusarium oxysporum*. Moreover, no antifungal activity was noted with MA14. However, with *Bacillus pumilus* strain MA9 and *Bacillus pumilus* strain MA19, the antifungal activity was highly effective against *Rhizoctonia solani* but less effective against *Fusarium solani* (Figure 3).

### 2.2. Biofilm and Exopolysaccharides Production In Vitro

**Figure 4:**
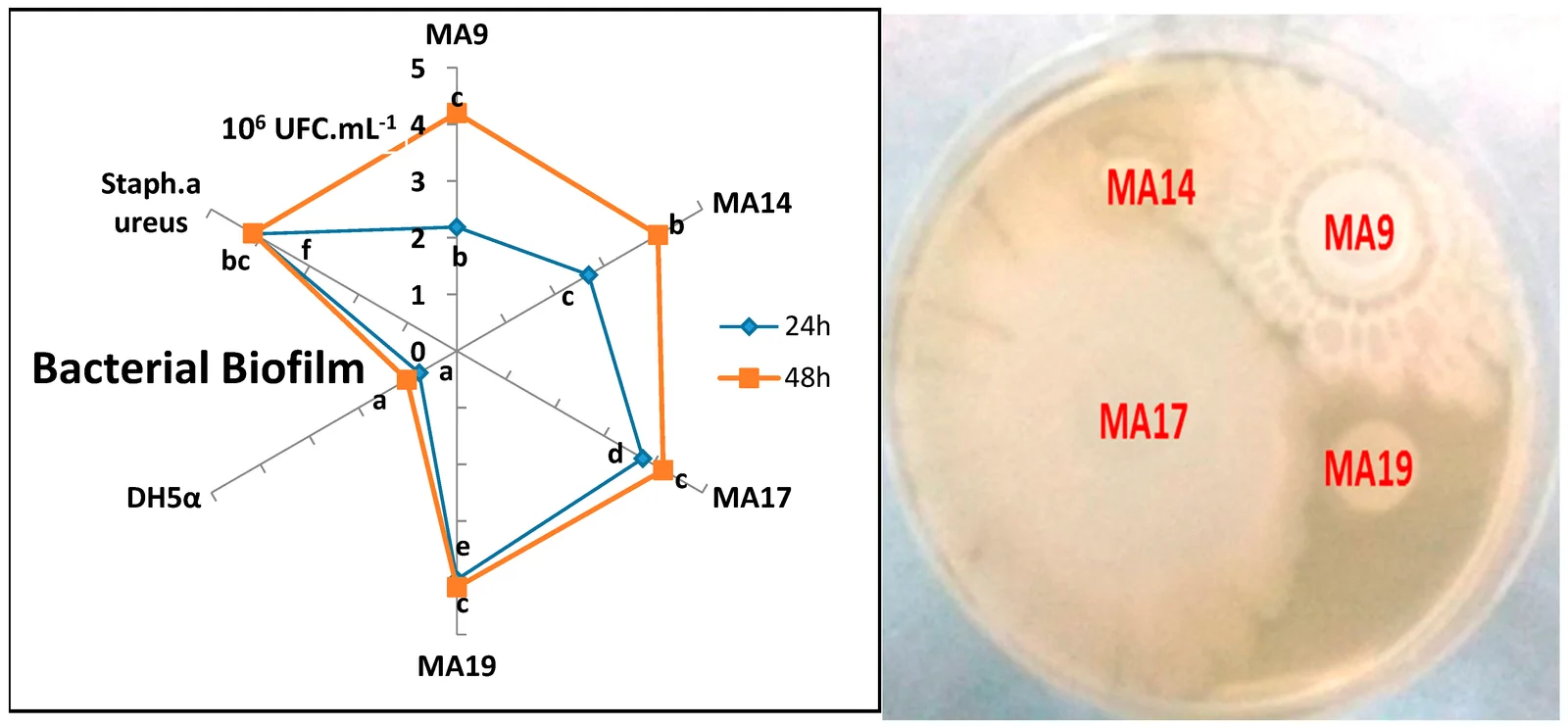
Biofilm formation and exopolysaccharides production of selected PGPB strains: (MA9) *Bacillus pumilus* strain MA9; (MA14) *Virgibacillus halodenitrificans* strain MA14; (MA17) *Bacillus subtilus* strain MA17, and (MA19) *Bacillus pumilus* strain MA19. Scale bars 50 µm. Different letters above the caption indicate a significant difference at *p* ˂ 0.05.

Biofilm production in vitro was studied using two control strains: *E. coli DH5α* (negative control) and *Staphylococcus aureus* sp. (positive control). After 24 h of incubation, the biofilm production capacity, compared with the two positive and negative controls, was more pronounced with *Bacillus subtilis* strain MA17 and *Bacillus pumilus* strain MA19 than with the other strains (Figure 4). However, the biofilm production capacities of *Bacillus pumilus* MA9 and *Virgibacillus halodenitrificans* MA14 were significantly higher compared to the negative control but lower than the biofilm production capacity of the positive control.

Exopolysaccharides production of selected PGPB strains (Figure 4) revealed that only the two strains, *Bacillus pumilus* strain MA9 and *Bacillus subtilis* strain MA17, produce EPS.

### 2.3. Effect of PGPB on the Morphology and Physiological Parameters of PGPB

The salt sensitivity assay showed that the durum wheat var. “aouija” seedling growth was affected at 40 mM and further severely retarded at 125 mM NaCl. Therefore, a 125 mM concentration was selected for all experiments. The present study revealed that durum wheat var. “aouija” could tolerate treated plantlets more effectively in hydroponic cultivation when bioprimed with halotolerant PGPB as opposed to the controls. In hydroponic culture without NaCl, all PGPB demonstrated improved plant development. When NaCl was introduced, unbioprimed plantlets saw severe effects on plant length, shoot length, and root elongation, but bioprimed plantlets showed less growth reduction. In the presence of *Virgibacillus halodenitrificans* strain MA14, *Bacillus subtilis* strain MA17, and *Bacillus pumilus* strain MA19, growth was significantly better than that of other strains (Table 1, Figure S3A). Although plants bioprimed with *Bacillus pumilus* strain MA9 did not exhibit appreciably higher growth, the latter was improved over control plantlets (Table 1). Under 0 mM, biomass plantlets bioprimed with all PGPB rose. However, at 125 mM, plantlets bioprimed with *Virgibacillus halodenitrificans* strain MA14, *Bacillus subtilis* strain MA17, and *Bacillus pumilus* strain MA19 showed a significantly higher biomass than MA9 (Table 1, Figure S3B).

Water leakage or variation in water retention reflected electrolyte leakage. Under stress and in the absence of any bacterial treatment, plants suffered from turgor and a reduction in ionic potential. Nevertheless, bacterial seed biopriming significantly improved water retention and thus reduced the leakage of electrolytes. The effect of strain on the reduction in intensity of the culture environment was more appreciable for treated plantlets than untreated plantlets, of which *Bacillus subtilis* strain MA17 presented the most dramatic percentage increase.

### 2.4. Effect of PGPB Biopriming on the Biochemical Parameters of Plants

**Figure 5:**
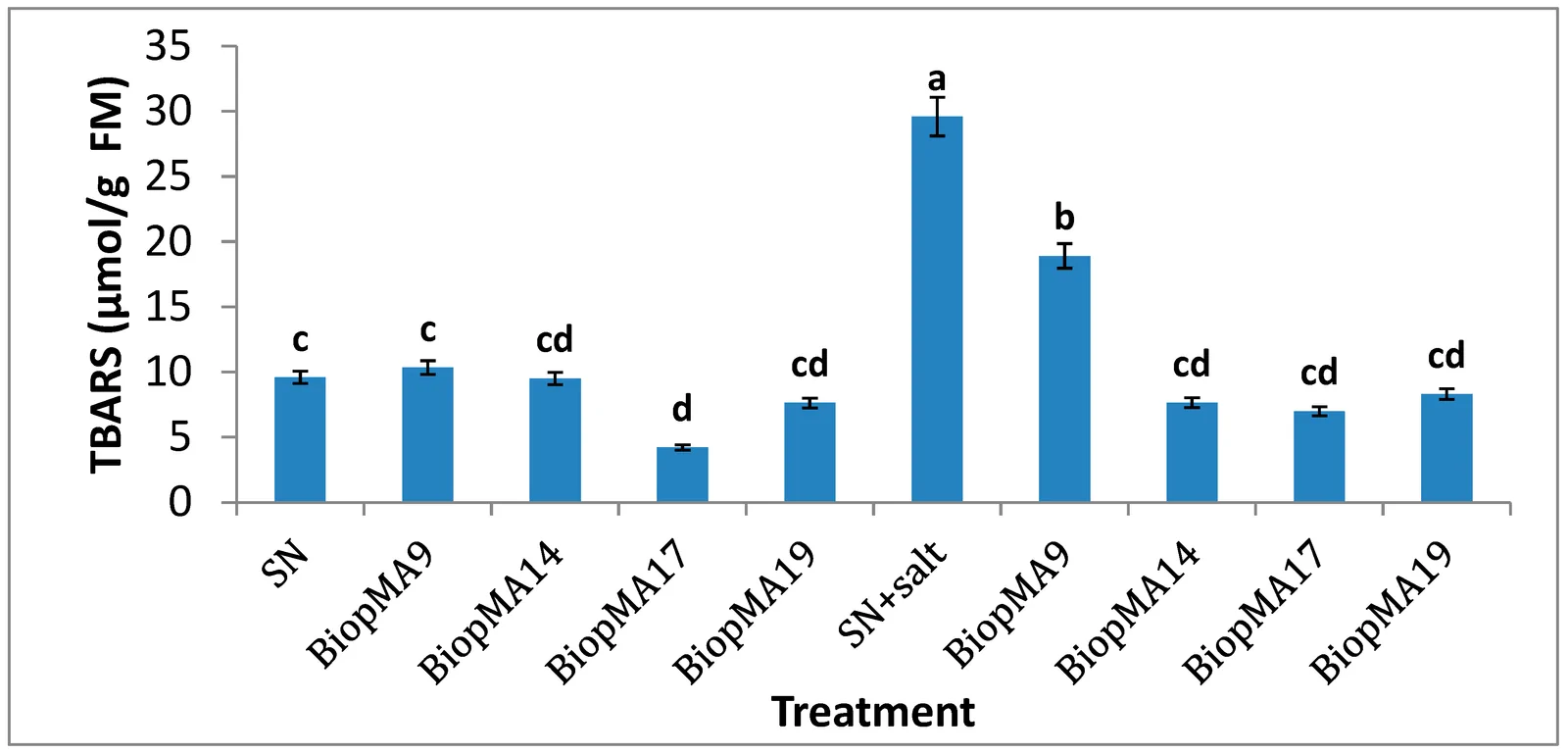
Seed biopriming with selected PGPB of durum wheat plantlets in terms of thiobarbituric acid reactive substances (TBARS) content (µmol/g FW) under nonsaline and saline conditions (in planta). Two Control: SN = untreated plantlets (0 mM); SN + NaCl = treated plantlets (125 mM); Biop = biopriming seed; MA9 = *Bacillus pumilus* MA9; MA14 = *Virgibacillus halodenitrificans* MA14; MA17 = *Bacillus subtilis* MA17: MA19 = *Bacillus safensis* MA19. Different letters above the error bars indicate a significant difference at *p* ˂ 0.05.

Analysis of lipid peroxidation revealed that the level of thiobarbituric acid reactive substances (TBARS) in untreated bioprimed plantlets with the different strains was decreased considerably compared with untreated plantlets (Figure 5). Results also showed that the *Bacillus subtilis* MA17 strain had the best ability to decrease the content of TBARS compared with the cultures bioprimed with other strains. In the case of biopriming alone, the *Bacillus subtilis* MA17 strain decreased the TBARS level by 76.12% compared with the control. Under stressful conditions, biopriming also had a significant effect on lowering the TBARS rate in treated plantlets.

**Figure 6:**
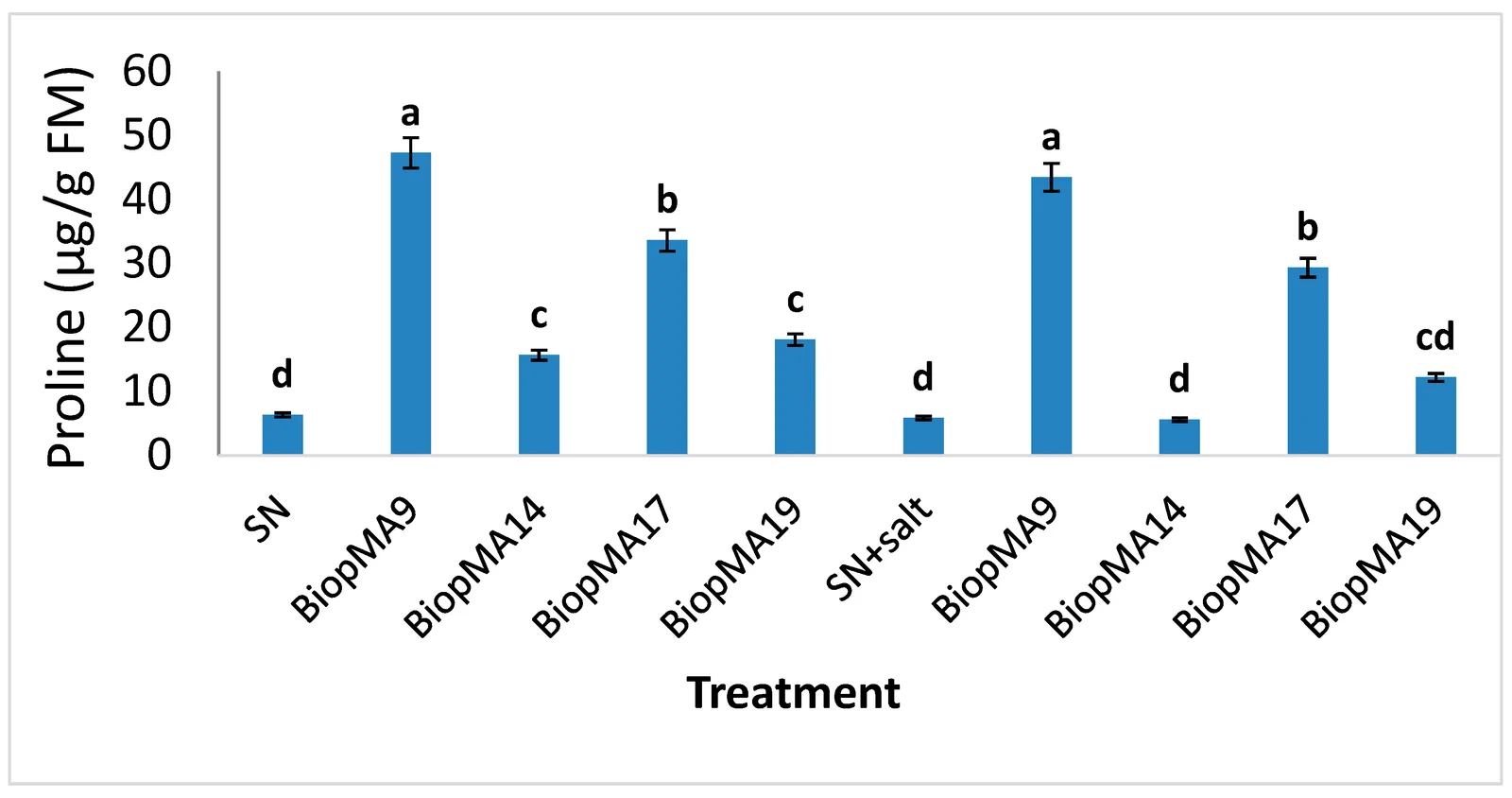
Seed biopriming with selected PGPB of durum wheat plantlets in terms of proline content (µg/g FW) under nonsaline and saline conditions (in planta). Two Control: SN = untreated plantlets (0 mM); SN + NaCl = treated plantlets (125 mM); Biop = biopriming seed; MA9 = *Bacillus pumilus* MA9; MA14 = *Virgibacillus halodenitrificans* MA14; MA17 = *Bacillus subtilis* MA17: MA19 = *Bacillus safensis* MA19. Different letters after the error bars indicate a significant difference at *p* ˂ 0.05.

Analysis of the leaf proline content in untreated plantlets showed that in the presence of PGPB, its concentration was significantly higher than the leaf proline content in the control. Results showed that the leaf proline content was strengthened by *Bacillus pumilus* MA9 and *Bacillus subtilis* MA17 strains, especially (Figure 6). Thus, biopriming makes it possible to increase proline synthesis both without and with saline stress. Furthermore, its production was considerably improved in salt-treated plantlets compared with untreated plantlets. This effect was most noticeable in *Bacillus pumilus* strain MA9, in which the leaf content of proline was quadrupled (from 12.93 μg/g FW to 53.6 μg/g FW).

**Figure 7:**
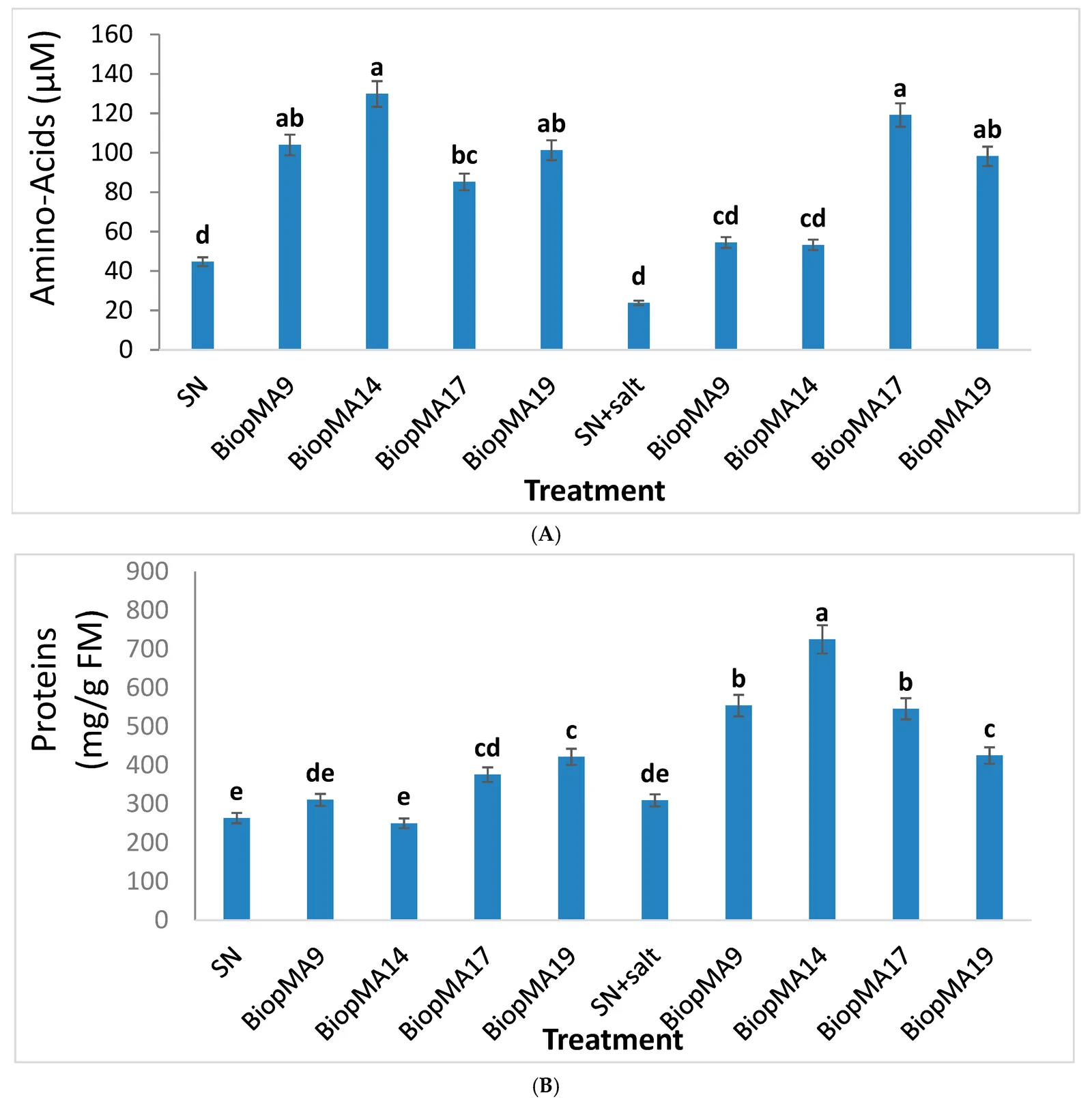
Seed biopriming with selected PGPB of durum wheat plantlets in terms of amino-acid (µM) (**A**) and protein content (**B**) (mg/g FW) under nonsaline and saline conditions (in planta). Two Control: SN = untreated plantlets (0 mM); SN + NaCl = treated plantlets (125 mM); Biop = biopriming seed; MA9 = *Bacillus pumilus* MA9; MA14 = *Virgibacillus halodenitrificans* MA14; MA17 = *Bacillus subtilis* MA17: MA19 = *Bacillus safensis* MA19. Different letters after the error bars indicate a significant difference at *p* ˂ 0.05.

The production rate of amino acid (AA) was more remarkable with the MA14 strain, in which the content of AA was tripled, compared to the untreated plantlets (Figure 7A).

The protein content was significantly reduced in treated plantlets in the absence of any bacterial treatment, whereas in the untreated culture, the protein level was normal. Protein expression was more pronounced in treated plantlets than in untreated plantlets, especially after biopriming with *Bacillus pumilus* MA9, *Virgibacillus halodenitrificans* MA14, and *Bacillus subtilis* MA17.

**Figure 8:**
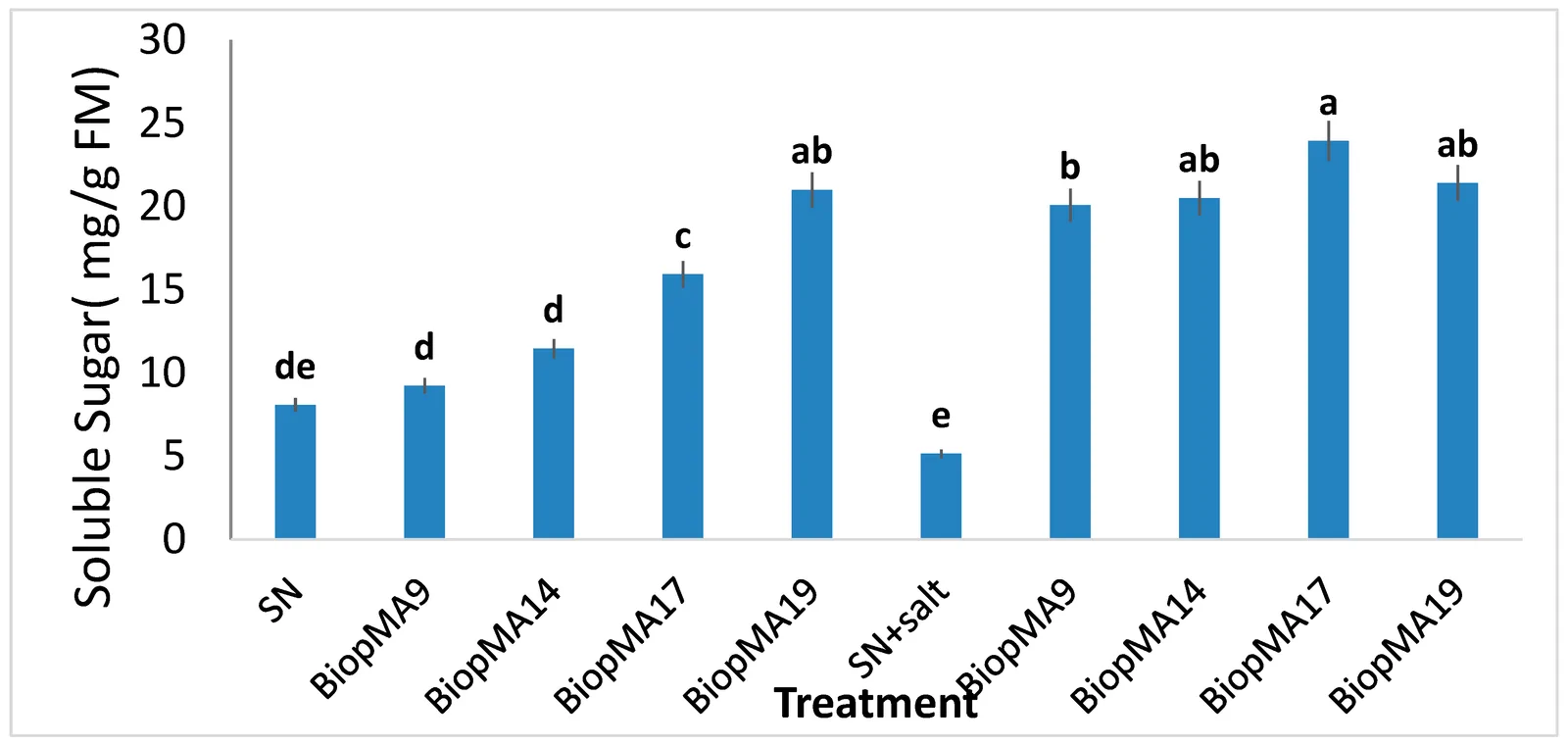
Seed biopriming with selected PGPB of durum wheat plantlets in terms of total sugar content (mg/g FW) under nonsaline and saline conditions (in planta). Two Control: SN = untreated plantlets (0 mM); SN + NaCl = treated plantlets (125 mM); Biop = biopriming seed; MA9 = *Bacillus pumilus* MA9; MA14 = *Virgibacillus halodenitrificans* MA14; MA17 = *Bacillus subtilis* MA17: MA19 = *Bacillus safensis* MA19. Different letters after the error bars indicate a significant difference at *p* ˂ 0.05.

The analysis of total sugars in plant tissue showed that an untreated bioprimed plantlet with potent strains had a significant effect on the total sugar content (Figure 8). However, this effect fluctuated according to the strain. Indeed, in the case of unbioprimed seed, the *Bacillus pumilus* MA19 strain provided the best result compared with the untreated plantlets. The strain with the best effect was, in this case, *Bacillus subtilis* strain MA17, which showed double the total sugar content. Under stressful conditions, the level of total sugars in bioprimed plantlets was considerably increased compared with the treated plantlets.

**Figure 9:**
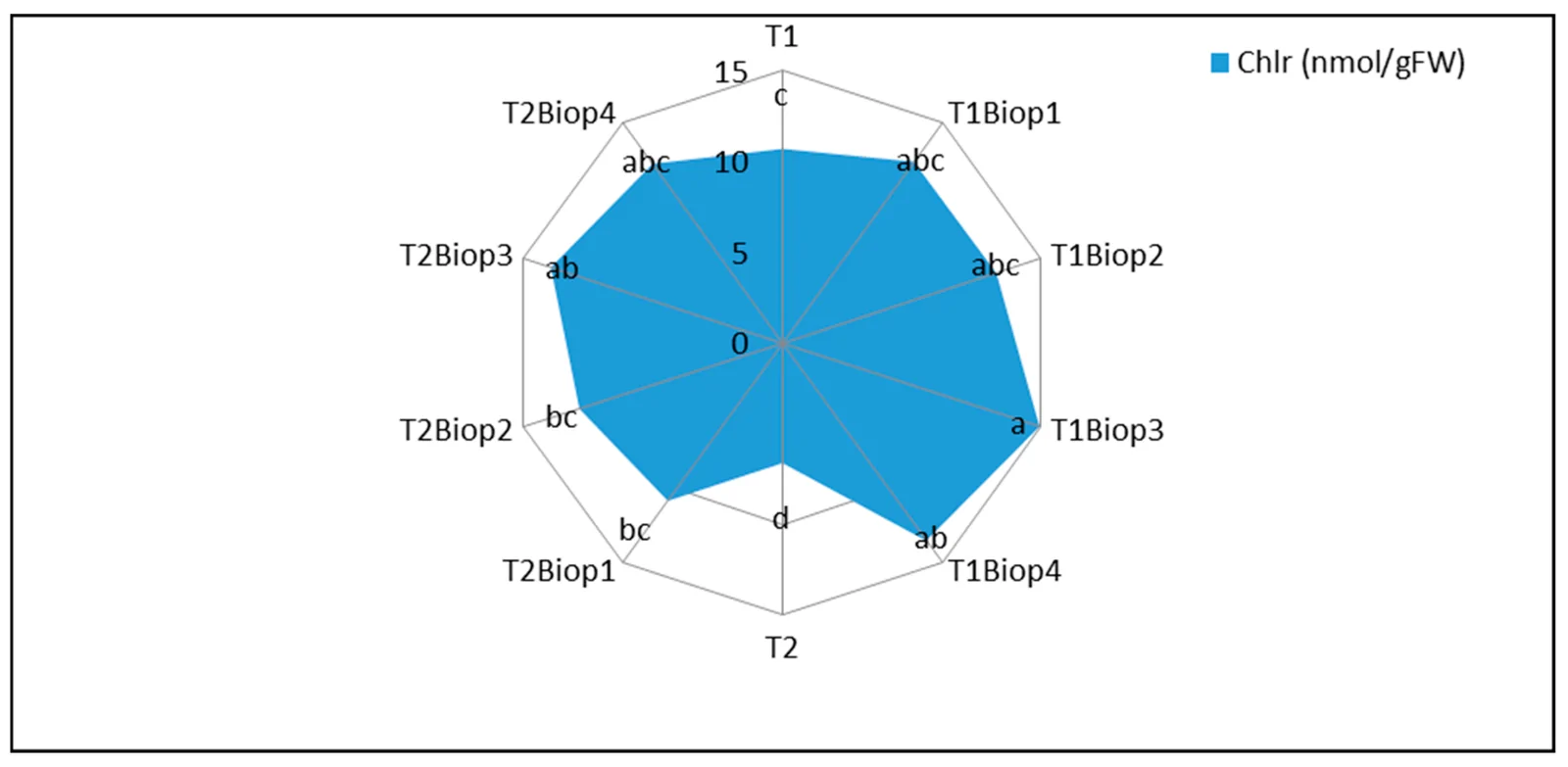
Seed biopriming with selected PGPB of durum wheat plantlets in terms of the chlorophyll pigment content (nmol/g FW) under nonsaline and saline conditions (in planta). Two Control: T1 = untreated plantlets (0 mM); T2 = treated plantlets (125 mM); Biop = biopriming seed; 1 = *Bacillus pumilus* MA9; 2 = *Virgibacillus halodenitrificans* MA14; 3 = *Bacillus subtilis* MA17: 4 = *Bacillus safensis* MA19. Different letters after the error bars indicate a significant difference at *p* ˂ 0.05.

The salt-treated plantlets (125 mM) strongly inhibited the formation of chlorophyll pigments. Biopriming with PGPB improved the chlorophyll pigment content in untreated as well as under-treated plantlets. Among the other strains, *Bacillus subtilis* strain MA17 still had the most productive effect on pigmentation under all conditions (Figure 9).

**Figure 10:**
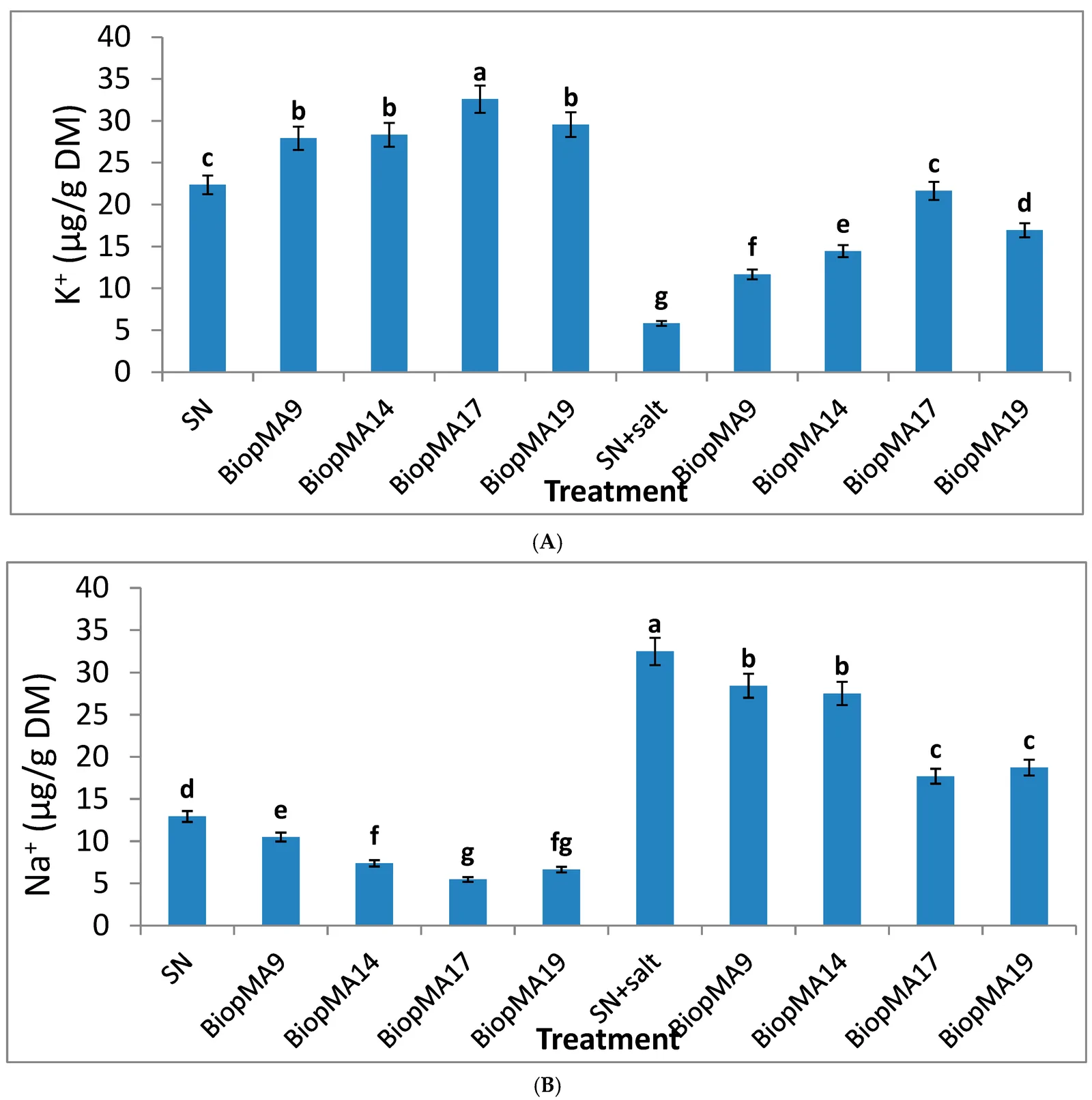
Seed biopriming with selected PGPB of durum wheat plantlets in terms of the ionic flux of K^+^ (**A**) and Na^+^ (**B**) (µg/g FW) under nonsaline and saline conditions (in planta). Two Control: SN = untreated plantlets (0 mM); SN + NaCl = treated plantlets (125 mM); Biop = biopriming seed; MA9 = *Bacillus pumilus* MA9; MA14 = *Virgibacillus halodenitrificans* MA14; MA17 = *Bacillus subtilis* MA17: MA19 = *Bacillus safensis* MA19. Different letters after the error bars indicate a significant difference at *p* ˂ 0.05.

In the present investigation, we found that the treated and bioprimed plantlets had a greater K^+^ content and a lower Na^+^ level. Therefore, because of limited Na^+^ uptake and increased K^+^ absorption, a greater K^+^/Na^+^ ratio was seen in plants. Unbioprimed plantlets had higher Na^+^ and lower K^+^ contents at 125 mM, while plants bioprimed with strains of *Bacillus subtilis* MA17 and *Bacillus pumilus* MA19 had the greatest decrease in Na^+^ concentrations. At both 0 and 125 mM, the K^+^ concentration and K^+^/Na^+^ ratio was greater in bioprimed plantlets with all PGPB (Figure 10A,B). Under salt stress, the K^+^/Na^+^ ratio of the bioprimed plants with PGPB was almost double that of the unbioprimed plants.

### 2.5. Expression of the Antioxidant Enzymatic System

**Figure 11:**
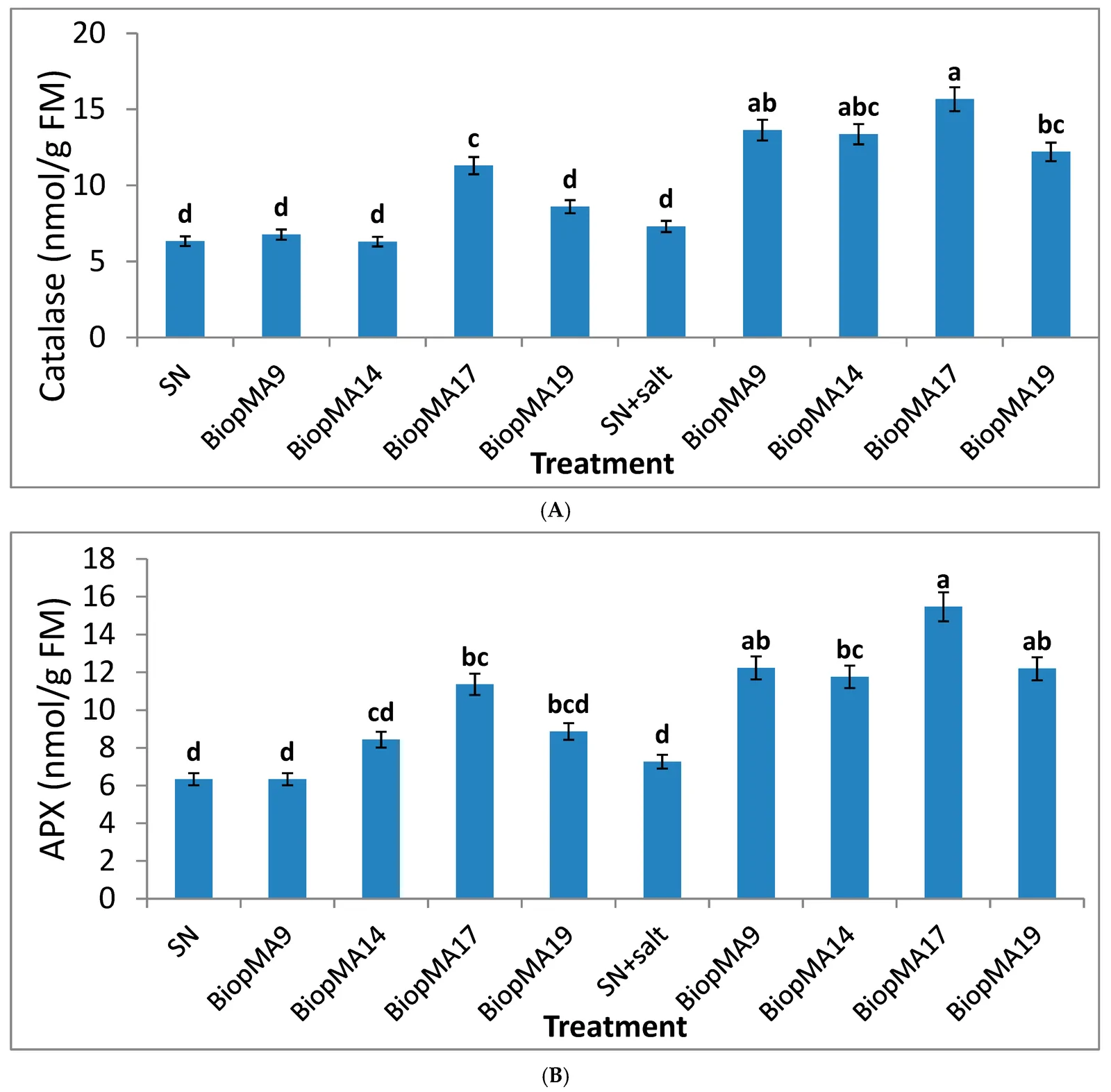
Seed biopriming with selected PGPB of durum wheat plantlets in terms of the content of the antioxidant enzymes catalase CAT (**A**) and ascorbate peroxidase APX (**B**) (nmol/g FW) under nonsaline and saline conditions (in planta). Two Control: SN = untreated plantlets (0 mM); SN + NaCl = treated plantlets (125 mM); Biop = biopriming seed; MA9 = *Bacillus pumilus* MA9; MA14 = *Virgibacillus halodenitrificans* MA14; MA17 = *Bacillus subtilis* MA17: MA19 = *Bacillus safensis* MA19. Different letters after the error bars indicate a significant difference at *p* ˂ 0.05.

Regardless of the microbiological treatment, plantlets that were not exposed to salinity-treated water exhibited comparable levels of catalase (Figure 11A) and ascorbate peroxidase activity (Figure 11B). Therefore, the seed-bioprimed plantlets showed increased levels of ascorbate peroxidase and catalase. Biopriming with these selected PGPBs significantly improved catalase activity in treated and untreated plantlets, and this significant effect was more pronounced in the *Bacillus subtilis* MA17 strain. Similarly, this significant effect of biopriming was observed for ascorbate peroxidase activity. The bioprimed plantlets showed elevated enzyme activities when compared to the control; however, no significant difference was found between the bacterial treatments, with the exception of the *Bacillus subtilis* MA17 strain.

## 3. Discussion

In this work, the positive effect of PGPB on the physiological and biochemical modifications of wheat plantlets under salt stress was studied. Many reports have underlined the function of *Virgibacillus* and *Bacillus* species, as well as *Exiguobacterium* species, in foresting plant development and reducing abiotic stress [38]. However, to the authors’ knowledge, this is the first study to evaluate the impact of seed biopriming with four *Bacillus* sp. strains on the physiological and biochemical pathways of Tunisian durum wheat var. “aouija” under stressful culture conditions in a controlled chamber. All the PGPB tested in this study were found to possess ACC deaminase, β-glucanase, and protease activity and produced copious amounts of exopolysaccharides, siderophores, and antifungal compounds, suggesting their possible role in reducing salt-induced damage in plants. Plant responses to salinity stress caused by NaCl have been extensively studied [39]. Under salt stress, plants maintain ion and osmotic homeostasis via ionic signaling perception and adjustment to rapid osmotic stress. Successful colonization establishment is the initial step for plant-bacteria interactions.

According to the present study, the selected strains had a significant power to form a bacterial biofilm in vitro after 24 h of growth (Figure 4), which indicated that these strains formed an adequate microenvironment for their growth and their strong colonization of the plant rhizosphere. Biofilm formation is assumed to be one such strategy for the bacterium to ascertain stable establishment on inert and living surfaces. However, biofilms play a potential role in enabling persistent microorganism establishment [40]. The selected strains used for seed biopriming exhibited many biological activities characterizing all PGPB (Table S1 and Figure 2 and Figure 3). Plant growth-promoting bacteria are recognized to reduce abiotic stress via a number of methods, including elevated proline level [10,41], mitigation of ethylene stress through ACC deaminase production [42,43,44], and exopolysaccharide-mediated reduction in Na^+^ uptake [40]. In addition, biofilm production after 48 h of incubation was the same in the four strains compared with the positive control. However, compared with the negative control, the ability to adhere to the surface remained stable for the four strains. For this reason, the *Bacillus subtilis* MA17 and *Bacillus pumilus* MA19 strains reached their biofilm production threshold faster than the other two strains.

In our study, Salt-treated plantlets decreased the growth medium’s osmotic potential and impacted water availability, resulting in physiological drought in plants. PGPB helps the plant by removing physiological drought under salt stress and increasing the water content in the cell [44]. Bioprimed plants (Figure S3) have a higher level of hydration, which improves water-use efficiency, boosts biomass, and enhances photosynthesis [45]. The cell’s membrane permeability is gauged by electrolyte leakage. Plant cells are damaged by salt, which makes them more permeable. The electrolytes in the membrane consequently seep out and build up in the surrounding tissues [46]. Electrolyte leakage from plant tissue was greatly reduced in PGPB-bioprimed plants with salt-treated plantlets in the current investigation, including that PGPB preserved the integrity of the plant cell membrane against the damaging effects of salt (Figure S3). A possible explanation for this discovery is that PGPB biopriming alters the saturation pattern of membrane phospholipids, which lowers membrane potential [41]. In comparison to unbioprimed plants, this study found that bioprimed plants had lower TBARS concentration due to PGPB. Therefore, PGPB not only decreased electrolyte leakage but also shielded plants from oxidative damage brought on by salt stress.

In the control plants, the percentage increase in water content was significantly lower, whereas bioprimed plants showed a higher percentage of water content (Figure S5). Analysis of the variances in water retention and electrolyte leakage capacity (Figure S4A,B) showed that these parameters were proportionately related. Indeed, in the absence of any bacterial treatment, the ability of the plants to overcome the turgor phenomenon decreased significantly with treated plantlets versus untreated plantlets. Thus, in salt-treated plantlets, seed biopriming with these bacteria significantly improved the potential of plants to retain water and further reduced the leakage of any essential nutrients, such as amino acids, sugars, and electrolytes. Thiobarbituric acid reactive substances (TBARS), such as Malondialdehyde (MDA), result from the decomposition of polyunsaturated fatty acids of cellular membranes and show a greater accumulation in salt-treated plantlets [47]. Consequently, MDA reflects the degree of the induced treated plantlets and the peroxidative damage brought on by reactive oxygen species [48]. Plant cell membranes are generally considered primary sites of salt injury. Membrane destabilization is frequently attributed to lipid peroxidation, which can be initiated by activated oxygen species, such as O^2−^, OH^−^, or _1_O^2^, or by the action of lipooxygenase [48]. The reduction in MDA concentration (Figure 5) in plants treated with PGPB in response to salinity is consistent with past research linking improved plantlets’ tolerance mechanisms to the control of lipid peroxidation [49]. Similar results have been reported for salt tolerance in various plants induced by PGPB [50,51].

Conversely, nonenzymatic antioxidants, such as cysteine, nonprotein thiol, proline, carotenoids, and ascorbic acid, may play a role in inducing resistance to salinity by protecting acid-labile macromolecules against attack by free radicals that are formed during various metabolic reactions leading to oxidative stress [52]. Interestingly, the plant’s natural salt mechanisms were linked to the increased proline concentration (Figure 6). According to Yoshiba et al. [53] and Mohamed and Gomaa [54], PGPB inoculation increased the proline content. This could be because the proline biosynthesis pathway is upregulated to maintain high proline levels. By preserving the cell water state, this phenomenon aids plants in salinity stress adaptation. Under osmotic stress, it has also been demonstrated that a large number of Gram-positive bacterial species increase the size of the proline pool and accumulate total free amino acids [50]. Proline and total soluble sugars are crucial biochemical markers of a plant’s ability to withstand salt [55,56]. Proline builds up in the cytosol of plants during salt stress and is crucial for cytoplasmic osmotic adjustment [57,58]. Additionally, proline supports the plant cell by scavenging free radicals, balancing the cellular redox potential during salt stress, and maintaining subcellular components, including membranes and proteins [59]. Proline, an amino acid, acts as a scavenger for ROS, and its accumulation at a well-defined concentration is a sign of stress [60].

Higher plants exposed to salt treatment acquire various amino acids and nitrogen-containing substances [61,62,63]. However, strain MA14, which showed the most potent effect under untreated plantlets, revealed a significant lowering of the total AA content under salt stress. Under stressful conditions, the AA content was also improved in the presence of PGPB compared with the control without biopriming. Moreover, under stress, the plants expressed many more proteins than the untreated plantlets (Figure 7B). The biological pathway of amino acid secretion was closely related to the total protein content in bioprimed plants with all the PGPB assessed (Figure 7A,B). In these two biological pathways, seed biopriming with the *Virgibacillus halodenitrificans* MA14 strain is more remarkable and pronounced under stress. This phenomenon can be explained by the marked transformation of the total AA into protein and can also be justified by the high proteolytic activity in this strain, which makes available the amino acids involved in protein chains and therefore their assimilation by the plant and their transformation into proteins. It has been found that the foliar sections of some salt-tolerant plants have large levels of total free amino acids [64,65]. A few of these proteins may have a direct correlation with salinity tolerance. Similarly, many proteins that are directly or indirectly related to plant growth promotion have been discovered to be expressed in rice plants through *Pseudomonas fluorescens* interaction [66].

The plant system’s ion homeostasis ratio becomes unbalanced due to salinity. Via scavenging in the vacuole utilizing NHX1 antiprotons or extrusion via the plasma membrane using the SOS pathway, plants attempt to maintain a low salt content in the cytosol. The cell’s Ca_2_^+^/K^+^ ratio is hampered by salinity. Nonetheless, the negative impact of salt on growth and yield can be mitigated by raising the K^+^ concentration [67]. Similarly, by decreasing the Na^+^ concentration and raising the K^+^ level, a number of PGPBs have been shown to lessen salt toxicity in a variety of different plants [68,69,70,71,72]. It is important to note that the effect of biopriming is more pronounced under salt stress. Hence, a clear difference was observed for the untreated plantlets of each strain compared with those exposed to stressful conditions (Figure 10A, B). For example, the two strains, *Bacillus subtilis* strain MA17 and *Bacillus pumilus* strain MA19, were twice as effective in treated than in untreated plantlets.

Catalases, superoxide dismutase (SOD), and ascorbate peroxidases were among the several ROS-scavenging enzymes that were part of the “ROS gene network”. When compared with the control, the bioprimed plants showed higher enzyme activity; nevertheless, there was no discernible difference between the bacterial treatments, except for MA17 (Figure 11A, B). The bacteria-bioprimed plants’ increased tolerance to salt stress may be linked to their higher levels of the enzymes catalase and ascorbate peroxidase [73,74,75,76].

These hydroponic results indicated that seed biopriming with these PGPB strains improved the tolerance induction, directly or indirectly, of physiological and biochemical pathways and therefore the tolerance of durum wheat “aouija” grown under salt stress conditions.

## 4. Materials and Methods

### 4.1. Microorganisms

The present study explores four salt-tolerant *Bacillus* spp. strains, namely, *Bacillus pumilus* strain MA9, *Virgibacillus halodenitrificans* strain MA14, *Bacillus subtilis* strain MA17, and *Bacillus pumilus* strain MA19, which were previously isolated from plant roots growing in coastal saline soil (*Salicornia brachiata*) in Tunisia, and screened for salt tolerance (ranging from 0 to 7 g/L) [12].

The 16S rRNA gene sequences of these strains have been submitted to GenBank under accession numbers MT672745, MT672746, MT672747, and MT672748 for *Bacillus pumilus* strain MA9, *Virgibacillus halodenitrificans* strain MA14, *Bacillus subtilis* strain MA17, and *Bacillus pumilus* strain MA19, respectively [12].

The cultures were maintained on trypticase soy agar under refrigerated conditions. The isolated strains exhibited PGP traits in vitro, such as IAA, phosphate solubilization, siderophore, HCN, antifungal, lytic enzymes, and ammonia production.

### 4.2. Biofilm and Exopolysaccharides Production

Strains from fresh agar plates were inoculated in 5 mL of tryptic soy broth and incubated for 24 h at 30 °C. Biofilm formation was conducted in 96-well flat-bottom plates (with lids) [77]. *E. coli* DH5*α* was used as a negative control, and *Staphylococcus aureus* M2 was used as a positive control. To visualize biofilm formation, the remaining bacteria attached to the bottom of wells were stained for 15 min at room temperature with 150 mL of 0.2% crystal violet solution prepared in 20% ethanol (*v*/*v*) [78]. Subsequently, crystal violet was eliminated, and the excess stain was rinsed three times with sterile water. The microplates were then inverted to remove any dye that was not staining the attached bacteria. Finally, 200 µL of glacial acetic acid was added to each well. The plates were incubated for 1 h at room temperature to promote biofilm bursting and solubilization of crystal violet that had already penetrated the cells. All tests were performed in triplicate. The OD of each well was measured at 570 nm using a microplate reader (Thermo Fisher Scientific Inc., Greenville, NC, USA).

The qualitative determination of exopolysaccharide production was performed according to [79]. Each strain was inoculated onto 5 mm diameter disks arranged in modified Burk medium: 2% yeast extract, 1.5% K_2_HPO_4_, 0.02% MgSO_4_, 0.0015% MnSO_4_, 0.0015% FeSO_4_, 0.003% CaCl_2_, 1.0% NaCl by addition of 10% sucrose. Production was characterized by the size of the halo produced and its viscous appearance. The production of EPS was confirmed by mixing a portion of the mucoid substance in 2 mL of absolute ethanol, where the formation of a precipitate indicated the presence of EPS [79].

### 4.3. Seed Biopriming with PGPB and Salt-Stress Treatment

Following a 0.1% HgCl_2_ surface sterilization, the seeds were rinsed four times with sterile bidistilled water. The PGPB utilized for seed biopriming was cultivated at 30 °C at 180 rpm for 24 h in DF salt media. 500 µL of primary culture was bioprimed in 50 mL of LB medium and cultivated to an OD 600 of 0.6at 30 °C at 180 rpm. After being collected for 10 min at 3550× *g* in GYROZEN 1580R Centrifuge, GYROZEN Co., Ltd., Daejeon, Republic of Korea, Cells were resuspended in MS medium at a concentration of 10^8^ CFU/mL^−1^. After being bioprimed by the diazotrophic PGPB chosen for this investigation, seeds of uniform size were put on sterile cotton in an autoclaved vial to germinate. A glass pot containing 50 mL MS major and minor components was filled with seven-day-old, germinated seedlings in four sets of ten plants each (0 mM NaCl) on a perforated Eppendorf. Five ml of the PGPB culture was used to bioprime two sets of Durum wheat plantlets. Two sets were subjected to 125 mM NaCl after four days of PGPB biopriming acclimation; the other group was not. Three replications of the bioprimed and control treatments were conducted. The plants were cultivated in a culture chamber with a light/dark cycle of 16 h/8 h at 25 ± 2 °C (350 mmol m^−2^ s^−1^ light intensity). After seven days, each pot’s medium was replaced. For both salt-treated and untreated plants, two pots devoid of PGPB inocula functioned as unbioprimed controls.

### 4.4. Sampling and Plant Analysis

The initial sampling is done after 21 days of cultivation. It constitutes the fresh samples for all subsequent analyses. After the beginning of the tillering stage (21-day culture), the growth parameters of the plants were observed. The fresh weight (FW) and total length of each plant were measured. Each plant was divided into roots and shoots, excluding the endosperm region, and the FW, length, and dry weight (DW) were recorded after drying in an oven at 70 °C for 72 h. The percentage increase in biomass was calculated using the following formula: % increase in biomass = (plant FW − seedling weight)/plant FW × 100.

#### 4.4.1. Electrolyte Leakage and Percentage Change in Water Content

Electrolyte leakage was measured according to [80]. One young leaf from the first primary branch toward the distal end of the same size was collected from three plants for each treatment and washed thoroughly with deionized water to remove surface-adhered electrolytes.

The samples were placed in closed vials containing 10 mL of deionized water and incubated at 25 °C on a rotary shaker for 24 h, and the electrical conductivity of the solution (Lt) was determined using Seven Easy conductivity (Metler Toledo AG, CH-8603 Schwerzenbach, Switzerland). Samples were then autoclaved at 120 °C for 20 min, and the final electrical conductivity (L_0_) was obtained after cooling at 25 °C. The electrolyte leakage was defined as follows:

Electrolyte leakage (%) = (L_t_/L_0_) × 100

The percentage change in water content was calculated as follows [81]:

[(FW − DW)/FW] × 100. Each experiment was replicated three times, and the mean values are provided in Figure 4.

#### 4.4.2. Peroxidation of Lipids

By measuring the concentration of thiobarbituric acid reactive substances (TBARS) generated by the thiobarbituric acid (TBA) reaction, lipid peroxidation was calculated in accordance with the method of Hodges et al. and [82]. In total, 15 mL of 80% alcohol was used to homogenize 0.5 g of leaf material, 1 mL of extract and 1 mL of 0.5% (*w*/*v*) TBA in 20% (*w*/*v*) TCA were combined in one set. TBA was not included in another set. After 30 min of incubation at 90 °C, the mixture was allowed to cool at ambient temperature. After centrifuging the samples for 3 min at 2147× *g* in the GYROZEN 1580R Centrifuge, GYROZEN Co. Ltd., Daejeon, Republic of Korea, the absorbance of the supernatant was measured at 400, 532, and 600 nm. The following formula was used to calculate the MDA concentration with:

A = [(Abs_532_ + TBA − Abs_600_ + TBA) − (Abs_532_ − TBA − Abs_600_ −TBA)]

B = [(Abs_440_ + TBA − Abs_600_ − TBA) 0.0571]

TBARS equivalents nmol g^−1^ FW = ([A − B]/157,000) × 10^6^

#### 4.4.3. Biochemical Analysis

With a few modest adjustments, ninhydrin was used to measure the amount of free proline in the leaves [83,84]. After homogenizing 100 mg of plant tissue in 1.2 mL of 3% aqueous sulfosalicylic acid, the samples were centrifuged for 10 min at 22,673× *g* (DAIHAN LabTech. Co., Ltd., Namyangju, Republic of Korea, LSI-3016A). Following centrifugation, 500 µL of supernatant was mixed with 1 mL of 2% ninhydrin and raised to 1 mL with distilled water. For 1 h, the mixture was incubated at 90 °C. After cooling the samples in an ice bath, 2 mL of toluene was added, and the mixture was vortexed for 2 min. In order to measure the absorbance at 520 using a VWR UV-1600 PC spectrophotometer, Scientific Instruments, Athens, Greece the upper phase was aliquoted in an Eppendorf tubes. By comparing the absorbance with a standard curve drawn from known concentrations of L-proline (Sigma Aldrich, St. Louis, MO, USA), the proline content was determined and represented as µg/mg FW.

With a few minor adjustments, the total amino acid content was calculated using the methodology outlined by [56,85]. After treating one milliliter of the plant extract with 1 mL of 0.2 M citrate buffer (pH 5), 1 mL of 80% ethanol, and 1 mL of ninhydrin (1%), the mixture was incubated for 15 min at 95 °C. A Jenway 7305 spectrophotometer, Bibby Scientific Ltd., Stone, Staffs, UK. was used to measure the absorbance at 570 nm [86] after the samples had cooled.

0.5 g homogenized plant tissue in 2 mL of extraction buffer [50 mM Tris–HCl (pH 8.3), 1 mM EDTA, 3 mM DTT, 0.08% ascorbic acid, 1 mM PMSF] was used to calculate the total protein content. The Bradford method was used to quantify the protein [87]. The total soluble sugars were analyzed by treating 0.1 mL of the alcoholic extract with 3 mL of freshly prepared anthrone reagent [150 mg anthrone in 100 mL of 72% (*v*/*v*) H_2_SO_4_] and placed in a boiling water bath for 10 min according to [85,88]. After cooling, the absorbance at 620 nm was determined using a UV–1600 PC spectrophotometer. The calibration curve was generated using glucose (Sigma Aldrich, USA) in the range from 20 to 400 µg/mL.

The chlorophyll content was determined according to the method of [89] modified by [90]. This method consists of cutting 0.5 g of fresh material from each sample into small segments, homogenizing them in 5 mL of 80% acetone, and leaving them in the freezer overnight. After filtration, the optical density was measured at 663 and 645 nm. The concentrations of chlorophyll **a** and **b** were deduced according to the following formula:

Chl**a** (mg/L) = 12.7 OD_663_ − 2.69 OD_645_;

Chl**b** (mg/L) = 22.9 OD_645_ − 4.68 OD_660_; Chl total = Chla + Chlb

where Chla is the concentration of chlorophyll a, Chlb is the concentration of chlorophyll b, and Chl total is the total chlorophyll concentration.

0.2 g of plant tissue was digested with 4 mL of perchloric acid and nitric acid solution (3:1) to determine the ion content. After being dried on a hot plate, the solution was diluted with deionized water to a volume of 25 mL and filtered through a 0.2 µm filter. An inductively coupled plasma optical emission spectrometer was used to evaluate the ion content [91].

#### 4.4.4. Antioxidant Enzymes of Plants

Using a mortar and pestle, fresh shoot and root samples (0.5 g) were homogenized in 5 mL of buffer (0.05 M Tris HCl buffer, pH = 7.5, 3 mM MgCl_2_, 1 mM EDTA), and centrifugation was performed in the GYROZEN 1580R Centrifuge, GYROZEN Co., Ltd., Daejeon, Republic of Korea. The APX and CAT activities were ascertained using the enzyme extracts that were so produced. The extraction buffer was supplemented with 2 mM ascorbate for determination of APX, in order to measure APX. A reaction mixture of 50 mM phosphate buffer, 0.2 mL H_2_O_2_ 1% (*w*/*v*), 0.5 mM ascorbate, and 0.1 mM EDTA was mixed with 0.1 mL of the enzyme extracts for measurement of APX. Furthermore, at 240 nm, a drop in ascorbate absorbance (extinction coefficient 2.8 mM^−1^ cm^−1^) was noted [92]. By adding 50 µL of enzyme extract to the reaction mixture that contained phosphate buffer and H_2_O_2,_ and then monitoring the drop in H_2_O_2_ absorbance (extinction coefficient 0.036 mM^−1^ cm^−1^) within 1 min at 240 nm, the activity of CAT was determined [93].

### 4.5. Statistical Analysis

The plant’s worth of data was recorded, and each experiment was conducted three times. Using IBM SPSS Statistics software v. 21.0 (SPSS Inc., Chicago, IL, USA), the data were examined for significance using analysis of variance followed by Duncan’s least significant difference test (*p* = 0.05). To identify significant differences between the means of different bacterial treatments under both control (0 mM) and 125 mM NaCl stress conditions, the critical difference (C.D.) values were calculated at the *p* = 0.05 level. Different letters represent significantly different mean values.

## 5. Conclusions

Seed biopriming with the *Bacillus subtilis* strain MA17 could protect wheat from salt stress more than the other tested strains. The alleviation of salt stress in wheat could be considered the integration of multiple physiological processes, including the improvement of plant mineral nutrition, increased plant antioxidative capacity and regulation of plant growth by plant hormones (IAAs), siderophores and lytic enzymes or activation of ion transporters leading to an increased ratio of K^+^/Na^+^ in wheat plants. These isolates might be useful in formulating new inoculants with combinations of different mechanisms of action, leading to a more efficient application for salt-treated plantlet adaptation and thereby improving cropping systems. Therefore, it is plausible that PGPB is an effective approach for improving the growth of salt-sensitive plants, a strategy that could be applied for sustainable agriculture.

## Figures and Tables

**Table 1:** Seed biopriming of durum wheat plantlets in terms of the percentage increase in biomass by selected PGPB on growth parameters (total length and total dry weight) under nonsaline and saline conditions (in vivo). Two Control SN = untreated plantlets (0 mM); SN + NaCl = treated plantlets (125 mM); Biop = biopriming seed; MA9 = *Bacillus pumilus* MA9; MA14 = *Virgibacillus halodenitrificans* MA14; MA17 = *Bacillus subtilis* MA17; MA19 = *Bacillus pumilus* MA19. Different letters after the values indicate a significant difference at *p* ˂ 0.05.

Treatment	Total Length (cm)	% Increase	Total Dry Mass (mg/Plant)	% Increase
SN	12.3333 de	-	53 bc	-
BiopMA9	22 b	78	55.3333 b	42
BiopMA14	13 d	05	46 d	18
BiopMA17	24.3333 a	97	45.6667 d	17
BiopMA19	24.3333 a	97	57 b	46
SN + salt	7.6667 g	**-**	24.3333 f	**-**
BiopMA9	10 f	30.5	65 a	167
BiopMA14	10.3333 ef	35	36 e	48
BiopMA17	19 c	148	51.3333 c	111
BiopMA19	14.3333 d	87	62 a	154.8

## Data Availability

The original contributions presented in this study are included in the article/Supplementary Materials. Further inquiries can be directed to the corresponding authors.

## Supplementary Materials

The following supporting information can be downloaded at: https://www.mdpi.com/article/10.3390/ijms27198889/s1.
