# Supplementary material for: Biopriming of Durum Wheat Seeds with Diazotrophic Bacteria Improved Physiological and Biochemical Pathways Under Salt Stress
Source: Int J Mol Sci. 2026 Oct 7;27(19):8889. doi: 10.3390/ijms27198889 (PMC13665189; doi:10.3390/ijms27198889)
Supplement: Supplementary file 1 [file ijms-27-08889-s001.zip › ijms-4162263-supplementary.pdf]

Table S1: Plant growth-promoting traits and other properties of selected isolates from coastal saline soil in Tunisia.

	Plant Growth Promotion Potentiel						Growth Mycelium Inhibition			
**Code**	**HCN**	**NH_3**	**Lytic Enzymes.**	**SDR**	**ACC deaminase***	**EPS**	***Rhiz. solani***	***Fus. solani***	***Fus. oxysporum***	***Fus. graminearum***
**MA9**	-	+++	++	++	+	++++	+++	-	-	+
**MA14**	-	++++	++	++++	++	+	-	-	-	-
**MA17**	-	++++	+++	++	++	++++	+++	+++	++	+++
**MA19**	-	+++	+++	+++	+++	+	+++	+++	++	+++

* ACC deaminase activity was measured after 7 days of bacterial growth. (+) low (++) medium (+++) high (++++) very high activity. All experiments were performed in duplicate.

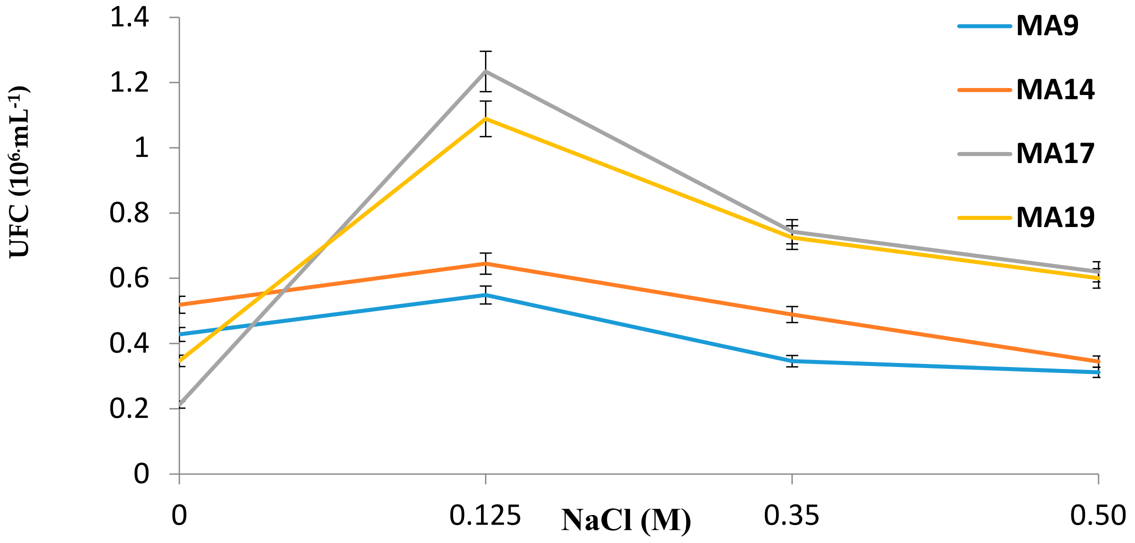

Figure S1: Effect of different concentrations of NaCl on the growth of selected strains (MA9, MA14, MA17 and MA19) on NFMM minimum medium at 30° C for 3 days. Error bars show the standard deviation of the mean values of three replicates ($p < 0.05$).

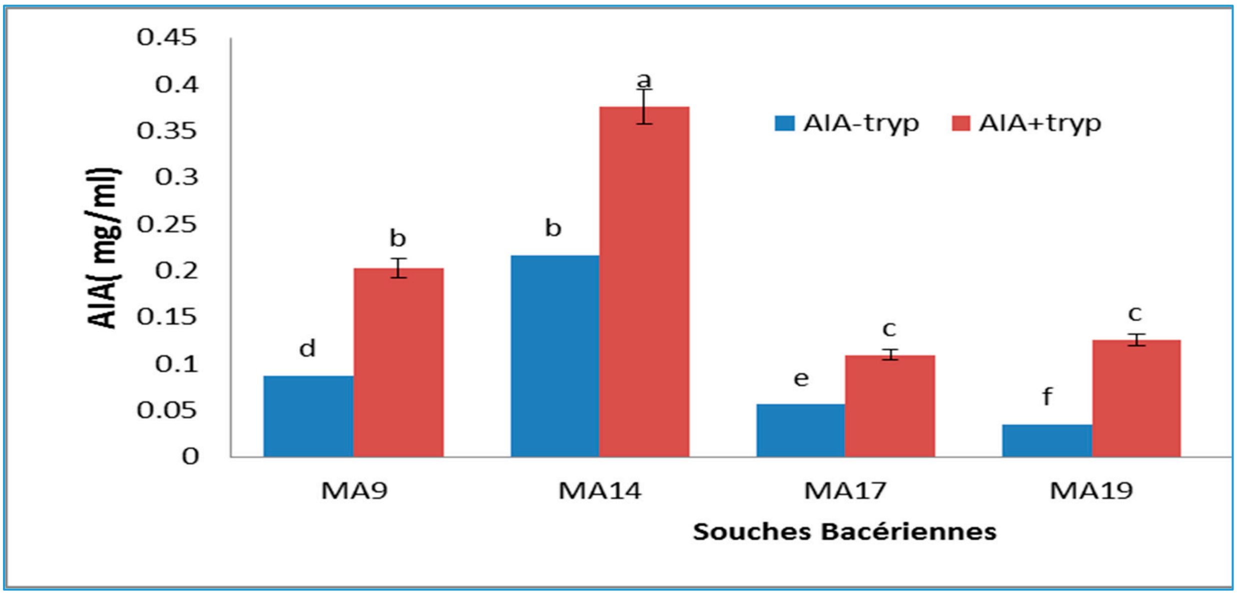

Figure S2: IAA production of selected strains on LB medium supplemented with 1% NaCl. Error bars indicate the standard deviation of the mean values of three replicates (P <0.05). Different letters after the values indicate a significant difference at $p < 0.05$.

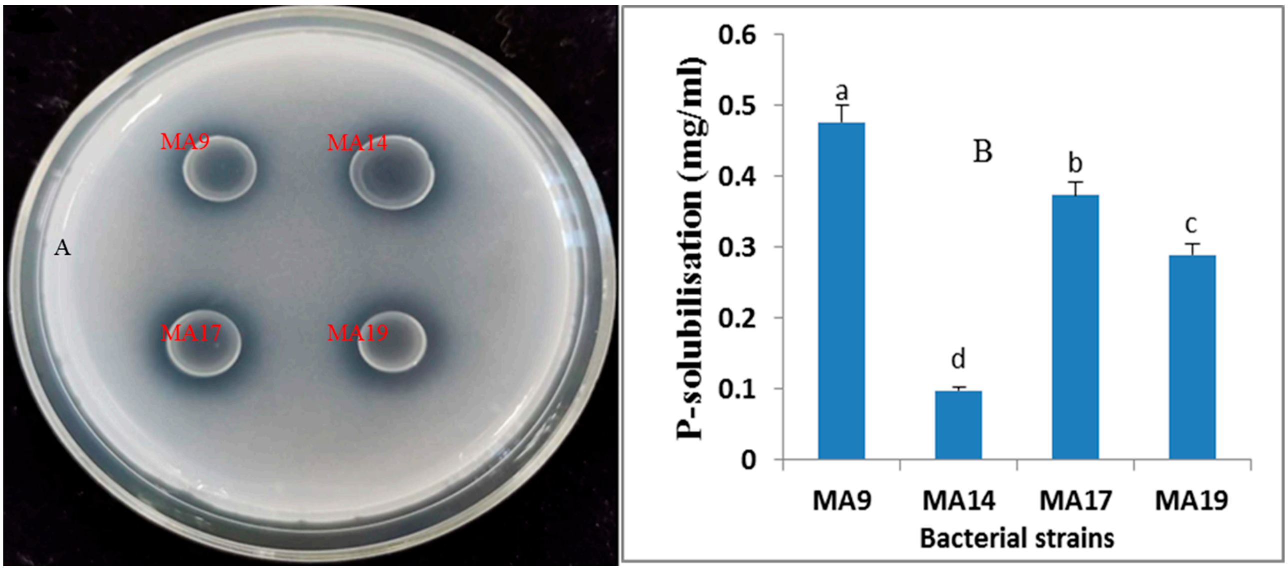

Figure S3: Inorganic phosphate (calcium) solubilization of selected strains on solid (A) and liquid (B) NBRIB medium with 1% NaCl. Error bars indicate standard deviation of mean values from three replicates (P <0.05). Different letters after the values indicate a significant difference at $p < 0.05$.

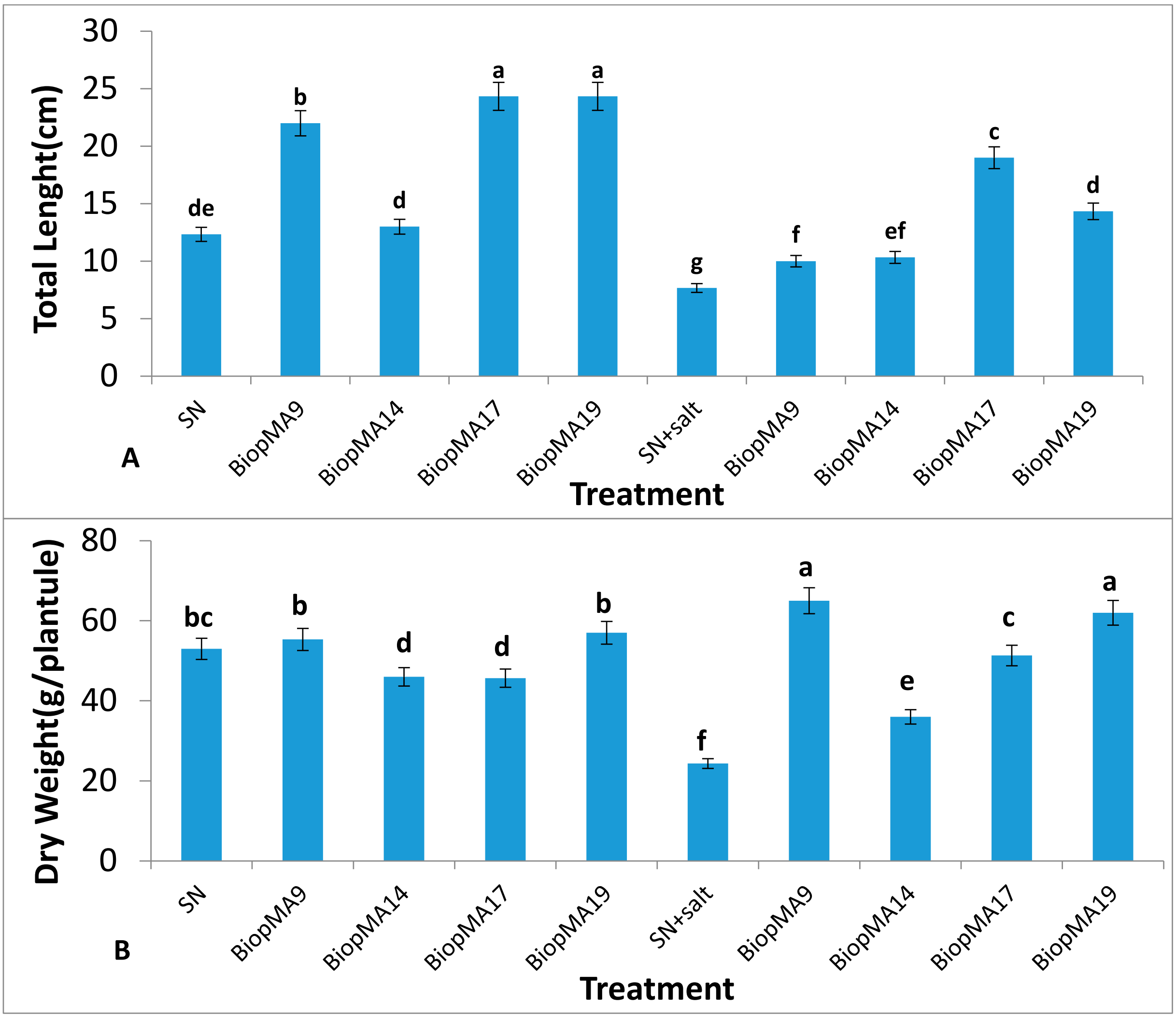

Figure S4: Effect of seed biopriming with PGPB on the total length (cm) (**A**) and total dry material (mg/plant) (**B**) of durum wheat plants under two salinity regimes, SN and SN+salt (0 and 125 mM). Two Control SN = untreated plantlets (0 mM); SN+ salt = treated plantlets (125 mM); Biop = biopriming seed with PGPB strains; MA9= *Bacillus pumilus* MA9; MA14= *Virgibacillus halodenitrificans* MA14; MA17= *Bacillus subtilis* MA17: MA19 = *Bacillus safensis* MA19. Different letters after the values indicate a significant difference at $p < 0.05$.

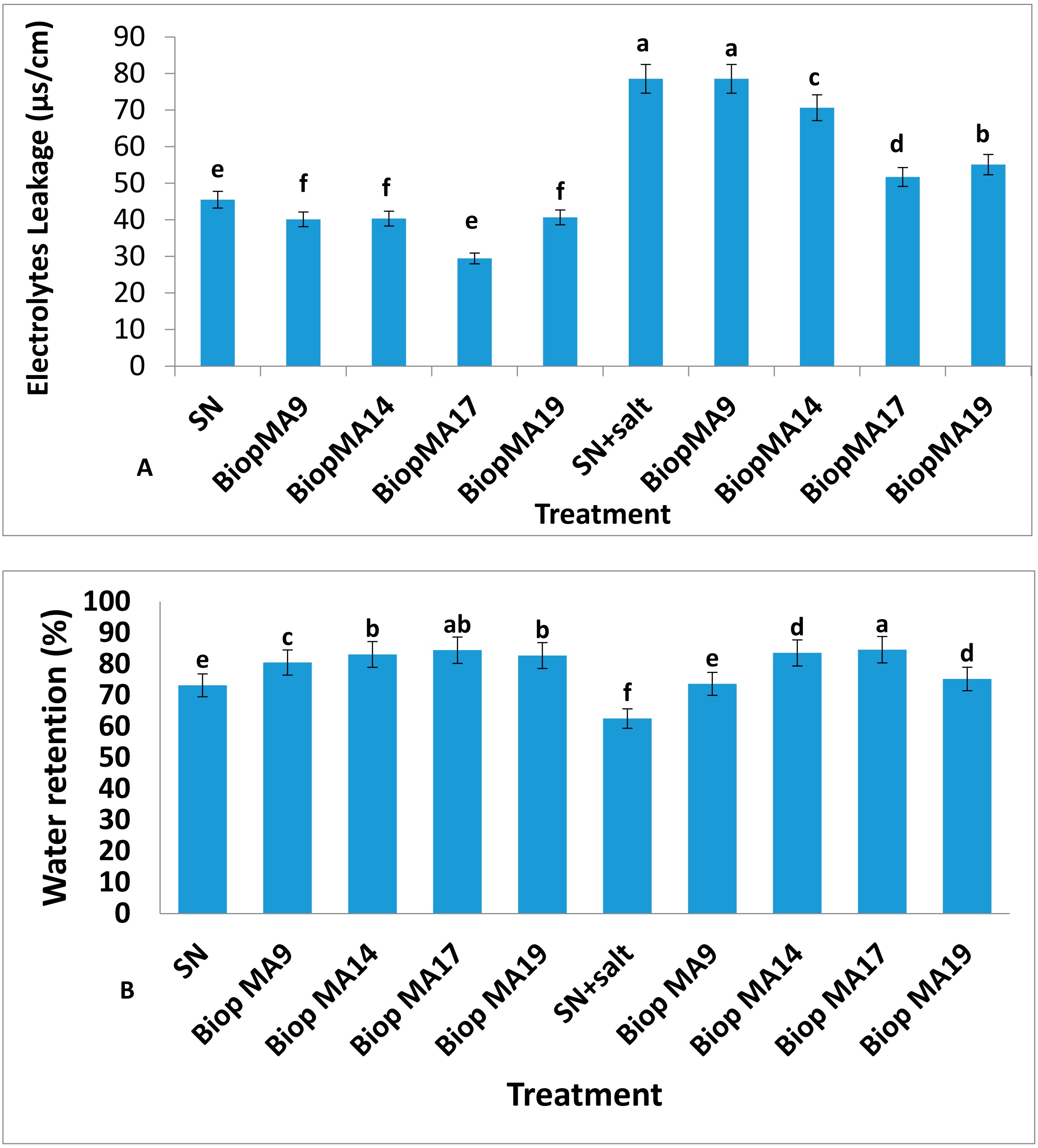

Figure S5: Effect of seed biopriming with PGPB on the electrolyte leakage (µs/cm) (**A**) and water retention (%) (**B**) of durum wheat plants under two salinity regimes, SN and SN+salt (0 and 125 mM). Two Control SN = untreated plantlets (0 mM); SN+ salt = treated plantlets (125 mM); Biop = biopriming seed with PGPB strains; MA9= *Bacillus pumilus* MA9; MA14= *Virgibacillus halodenitrificans* MA14; MA17= *Bacillus subtilis*

MA17: MA19 = *Bacillus safensis* MA19. Different letters after the values indicate a significant difference at $p < 0.05$.
